# Methods of Surgical Repair for Iatrogenic Sigmoid Colon Perforation Following Colonoscopy: A Case Report and Literature Review

**DOI:** 10.7759/cureus.47346

**Published:** 2023-10-19

**Authors:** Hannah W Weinstein, Shamon Gumbs, Sharique Nazir

**Affiliations:** 1 General Surgery, Columbia University College of Physicians and Surgeons, Harlem Hospital Center, New York, USA; 2 Minimally Invasive Surgery/General Surgery, NYC Health + Hospitals/Harlem, New York, USA

**Keywords:** surgical emergencies, post-colonoscopy colon perforation, iatrogenic colonic perforation, imatinib, colonoscopy, repair iatrogenic colon injury, surgical management colonic perforation, sigmoid colon perforation

## Abstract

Iatrogenic colonic perforation is a relatively infrequent yet perilous complication arising from both diagnostic and therapeutic colonoscopies, potentially leading to severe septic complications and increased morbidity or mortality. Given the gravity of potential complications, surgical intervention stands as the principal treatment strategy, with various modalities selected based on clinical discretion. In this context, we present the case of a patient who underwent primary laparoscopic repair following the identification of a sigmoid colon perforation during a routine colonoscopy. Intraoperatively, a Jackson-Pratt drain was placed to facilitate postoperative monitoring and drainage. The patient's hospitalization extended to a total of seven days due to sustained drainage and leukocytosis, highlighting the complexities of managing postoperative complications in such cases. This report underscores the current landscape of published data guiding the surgical management of iatrogenic colonic perforation following colonoscopy and highlights both the existing strengths and gaps within the current body of literature. As colonic perforation remains a critical concern in endoscopic procedures, a comprehensive understanding of optimal surgical interventions is crucial for minimizing patient morbidity and ensuring successful outcomes.

## Introduction

Iatrogenic perforation of the sigmoid colon is a rare yet pernicious complication of diagnostic or therapeutic colonoscopy. The total incidence of iatrogenic perforation following colonoscopy is estimated between 0.032% and 0.15% across retrospective studies of large patient cohorts [1-6]. Perforation occurs at higher rates during therapeutic colonoscopy as compared to diagnostic colonoscopy, 0.02-8% and 0.016-0.8%, respectively [3,4,6]. The most common site of perforation is the sigmoid colon or rectosigmoid junction with studies reporting as high as 70% of perforations occurring at these anatomic locations [2,3]. Complications of perforation include peritonitis, sepsis, and death [7]. Given the urgency of iatrogenic colonic perforation, surgical repair is often necessary, although conservative management is a consideration. Surgical treatment options include endoscopic repair, laparoscopic repair, robot-assisted repair, or open surgery. To date, the chosen method of surgical repair is variable, depending on the surgeon and case presentation, including perforation location, size, and hemodynamic stability of the patient.

Herein, we describe the emergent surgical repair of a sigmoid colon perforation following a colonoscopy in a 67-year-old patient. This case report summarizes the preoperative considerations, the most common interventions, and the postoperative complications associated with the surgical management of iatrogenic perforation of the sigmoid colon.

## Case presentation

A 67-year-old female with a history of chronic myelogenous leukemia (CML) treated with imatinib (last dose the day prior to colonoscopy), gastritis, hyperlipidemia, hypertension, and three classic cesarean sections presented for a routine colonoscopy.

**Figure 1:**
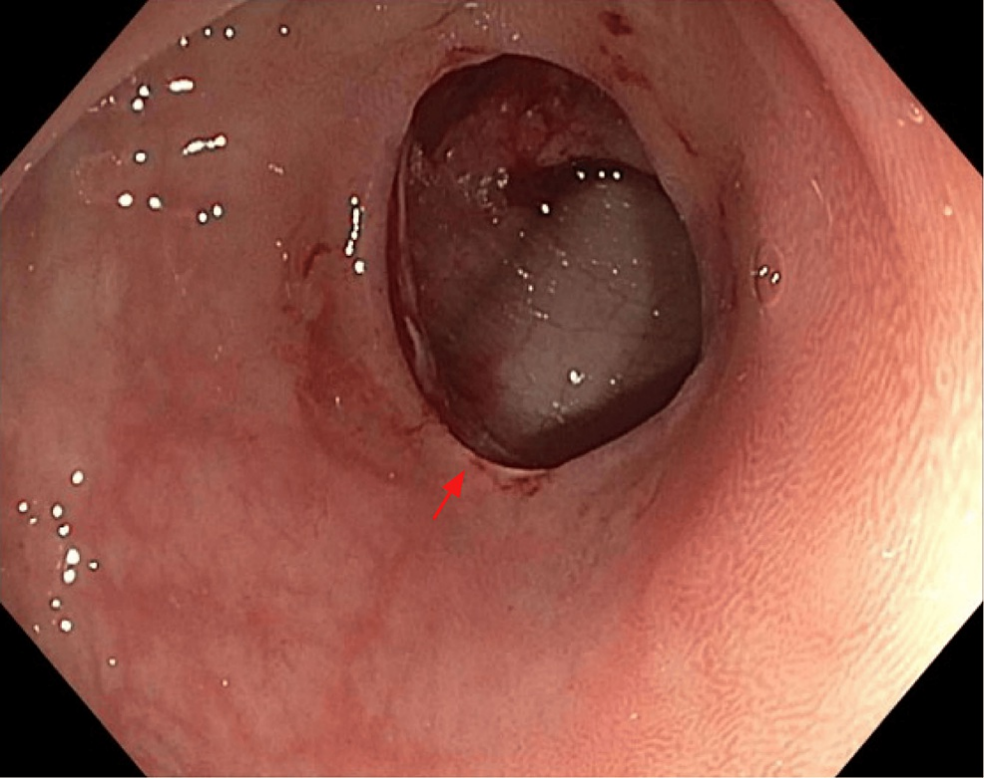
Colonoscopy visualization of the sigmoid colon demonstrating perforation

**Figure 2:**
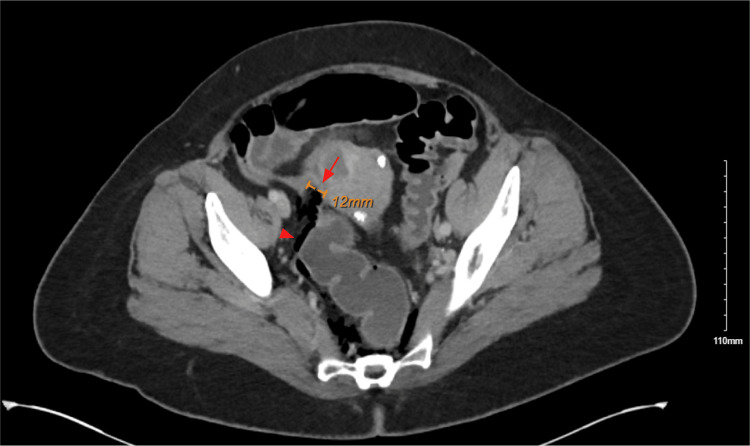
Preoperative CT of the abdomen/pelvis demonstrating sigmoid perforation and free intraperitoneal air

During the colonoscopy, there was difficulty navigating past the sigmoid colon, and then a perforation of the sigmoid colon was identified (Figure 1). General Surgery was consulted, and an abdominal CT was obtained given the patient was asymptomatic and hemodynamically stable. Abdominal CT with IV contrast revealed a 1.2 cm defect at the superior wall of the sigmoid loop, with adjacent extraluminal air extending into the intraperitoneal and extraperitoneal spaces, including the perirectal and retroperitoneal spaces (Figure 2). A few filling defects measuring up to 5 mm were present along the dependent rectal wall.

The patient underwent an emergent diagnostic laparoscopy. Intraoperative findings included a 1.2 cm sigmoid perforation near the rectosigmoid junction, approximately 30 mL of feculent fluid, fibrinous exudate, and evidence of retroperitoneal pneumatic dissection. A primary two-layer repair of the sigmoid perforation was performed with simple interrupted 2-0 silk sutures, followed by 2-0 silk Lembert-style sutures. Following abdominal washout, Gastroenterology then performed an intraoperative sigmoidoscopy and a leak test, with no signs of evident leakage. A Jackson-Pratt (JP) drain was placed in the pelvis extending along the left paracolic gutter and exiting through one of the 5 mm port sites. 

The postoperative course was remarkable for mild generalized abdominal tenderness and distension. The JP drain produced over 150 mL of serous drainage per day for five consecutive days. Laboratory findings were remarkable for leukocytosis that slowly declined. The patient was discharged with the JP drain in place on postoperative day 7 and treated in total with a 14-day course of antibiotics, seven days of IV piperacillin-tazobactam and then seven days of oral amoxicillin-sulbactam, due to concern for persistent infection in the setting of imatinib-induced immunosuppression. One week postoperatively, she followed up in the clinic, and the JP drain was removed. The patient was also seen three weeks post discharge, with good postoperative recovery.

## Discussion

The mean age for iatrogenic perforation of the sigmoid colon is 70 years with a female predominance [3]. Risk factors documented across multiple studies include advanced age (>65 years), low BMI, low albumin level, history of abdominal surgery, comorbidities including Crohn's disease and diverticulosis, experience of the endoscopist, and low volume centers [6]. Of note, spontaneous colonic perforation has been reported in the setting of imatinib, a tyrosine kinase inhibitor employed for various malignancies, including CML, as seen for this patient [8]. Perforation as a result of using imatinib is very rare with incidence not reported; as for the other tyrosine kinase inhibitors, some have an incidence of intestinal perforation of up to 4% (such as sorafenib). The exact mechanism of molecular targeted therapy-associated bowel perforation is unknown [9].

Approximately 45-60% of iatrogenic colon perforations are detected by the endoscopist during the procedure, as in this case, with the most common presenting symptoms including abdominal pain, rebound tenderness, abdominal distension, tachycardia, leukocytosis, and perirectal bleeding [2,3,6,10]. Very few patients (approximately 5%) are asymptomatic [3,6]. In a large retrospective study of over 50,000 colonoscopies, the average time following perforation to diagnosis was reported as 6.9 hours following a diagnostic procedure and 16.8 hours following a therapeutic procedure [3]. The morbidity and mortality rates in iatrogenic colon perforations are reported between 45-48.7% and 15-25.6%, respectively, from retrospective cohort studies [1-3]. The mean hospital stay is typically between eight and 15 days [2,4].

In 2017, the World Society of Emergency Surgery (WSES) released the first recommendations for the management of iatrogenic colonic perforation to provide a global standard [6]. For patients with localized pain, hemodynamic stability, no free fluid on radiographs, and small, sealed-off perforations, conservative management with serial examination, imaging, antibiotics, and bowel rest is appropriate [6]. Generally, colon perforations <50% circumference of the colon can be repaired primarily. Perforations <1 cm can be repaired endoscopically, whereas those >1 cm would require surgical intervention [4]. 

Endoscopic repair is recommended when perforation is identified intraprocedurally or within four hours following colonoscopy and can be conducted using endoscopic clip closure and/or endoloops [2,5,6]. High clinical success rates (57-100%) have been reported for these novel devices over the last several years [6].

Laparoscopic surgical repair stands as a safe and effective approach for addressing iatrogenic colonic perforation, currently reigning as the preferred intervention. Most laparoscopic repairs are done via primary repair with a simple suture [2,4]. Additional options include resection with anastomosis or, less frequently, Hartman's procedure [2]. Resection with primary anastomosis can be done when the perforation is >50% intestinal circumference, while Hartman's procedure can be done for delayed surgical intervention, gross intraperitoneal contamination, or hemodynamic instability [6,11]. Retrospective studies report bowel resection in up to 30% of cases [2,3]. Laparoscopic repair with an endoscopic linear stapler has also been successfully used in at least 14 patients [12].

A comprehensive meta-analysis involving 11 non-randomized controlled trials has yielded intriguing insights into the comparison of open and laparoscopic repair [7]. This analysis unveils a notable absence of discernible differences in critical parameters, including the intended colonoscopy objective, history of prior abdominopelvic surgery, perforation dimensions, and operative duration between these distinct surgical strategies [7]. However, compared to laparotomy, laparoscopic repair is associated with shorter incisions, hospital stays, postoperative fasting, recovery, and lower morbidity [4,7,13]. Nonetheless, it is noteworthy that approximately 10% of laparoscopic repairs necessitate conversion to open surgery [13]. The utilization of laparotomy is recommended when laparoscopic completion of the procedure becomes unfeasible [6]. Its predominant utilization comes into play for instances of substantial perforations, extensive peritoneal contamination, and impaired tissue viability or in situations where the patient's hemodynamic stability is compromised [6]. 

Robot-assisted surgical management for elective operations is a state-of-the-art practice, but the data on its use in the emergent operative space is minimal [14]. Per our search, the literature for robot-assisted repair of colonic perforation includes one case report of successful robotic primary colon repair using the da Vinci robot (Intuitive Surgical, Inc., Sunnyvale, California, United States) [15]. This case was undertaken using robotic technology to surpass the constraints of the laparoscopic approach, thereby offering enhanced visualization and precise maneuvers tailored to the unique requirements of the patient [15]. The perforation was closed via primary repair in 135 minutes, and the patient was discharged in four days [15].

Intraoperative preventative measures in the event of postoperative anastomotic leak may include the creation of a proximal diverting stoma. While there is no specific literature on drainage following surgical management of iatrogenic colonic perforation, the 2017 WSES guidelines recommend drainage in the setting of suspected peritoneal contamination, if bleeding or leakage from the repair is of concern, or for surgeries performed over 24 hours following perforation [6]. Ultimately, the decision is left to the responsible surgeon. Much like drainage, stoma formation lacks substantial evidence, yet it remains a viable choice carried out through surgical assessment most often in cases involving pronounced peritoneal contamination or the existence of malignant colonic neoplasms [1,6].

## Conclusions

Iatrogenic colonic perforation, although rare, presents a potentially urgent complication arising from both diagnostic and therapeutic colonoscopy. The prevailing approach to management involves prompt surgical intervention through laparoscopic repair. Although randomized clinical trials in this area are limited, the landscape of guidance for addressing iatrogenic colonic perforation is expanding, with increasing evidence outlining precise decision algorithms. Throughout this process, surgical expertise continues to play a pivotal role in shaping the treatment strategy. In light of the complexities associated with this condition, fostering ongoing research efforts remains essential to refine and optimize the management of iatrogenic colonic perforations.
